# Tolerance and adaptation characteristics of sugar beet (*Beta vulgaris* L.) to low nitrogen supply

**DOI:** 10.1080/15592324.2022.2159155

**Published:** 2022-12-25

**Authors:** Jiajia Li, Xinyu Liu, Qi Yao, Lingqing Xu, Wangsheng Li, Wenbo Tan, Qiuhong Wang, Wang Xing, Dali Liu

**Affiliations:** a National Beet Medium-Term Gene Bank, Heilongjiang University, Harbin 150080, P. R. China; b Key Laboratory of Sugar Beet Genetics and Breeding, Heilongjiang Province Common College/College of Advanced Agriculture and Ecological Environment, Heilongjiang University, Harbin 150080, P. R. China; c Key Laboratory of Molecular Biology, School of Life Sciences, Heilongjiang University, Harbin 150080, P. R. China

**Keywords:** Sugar beet, low nitrogen (LN), growth indexes, root morphology, antioxidant enzyme activity, comprehensive evaluation

## Abstract

Nitrogen (N) is an essential element required for sugar beet growth. Sugar beets with low N (LN) tolerance and high N use efficiency are excellent materials for breeding. Here, we comprehensively evaluated the morphological and physiological responses of nine sugar beet genotypes to LN supply. It was found that 0.5 mmol·L^−1^ N (LN) significantly influenced the performance of leaves and the topology of roots by reducing the bioproduction of chlorophyll a (Chl a) and soluble protein (SP) and the accumulation of N in leaves and roots (LNA and RNA), thus differentially restricting the growth (hypocotyl diameter, HD; root length, RL) and biomass (leaf and root fresh weight; LFW and RFW; leaf dry weight, LDW) of these sugar beets. Principal component and cluster analyses showed that 780016B/12 superior (F) exhibited excellent tolerance to LN; it had higher SOD activity (62.70%) and APX activity (188.92%) and a higher proline content (131.82%) than 92011 (G, LN sensitive). These attributes helped 780016B/12 superior (F) to better endure LN stress, and the morphology and N distribution changed to adapt to N deficiency, such that the root length increased by 112.48%, leaf area increased by 101.23%, and leaf nitrogen accumulation reached a peak of 14.13 g/plant. It seems that LN-tolerant genotypes increased their root length and surface area by reducing the difference in biomass, thereby expanding the contact between roots and soil, which was conducive to the absorption of nutrients (N) by sugar beets and helped distribute more assimilation products to the roots.

## 1 Introduction

Sugar beet (*Beta vulgaris* L.) is the most abundant sugar-producing crop species in northern China.^1^ Its sugar production and consumption rank among the highest in the world.^2^ Sugar beet accounts for 20% of the total sugar production worldwide, while the remaining 80% comes from sugarcane.^3^ However, sugar beet is superior to sugarcane in terms of sucrose content, which is usually 13–20% in beetroot.^4,5^ There are many factors limiting the growth and development of sugar beet, including nutrient deficiency, especially that of nitrogen (N). To protect the soil and biodiversity, as well as obtain high yields and high-quality sugar, it is necessary to optimize fertilizer inputs in sugar beet production.

N is an essential component of proteins, nucleic acids, chlorophyll, coenzymes, plant hormones, secondary metabolites, etc.^6^ N uptake is the first step in N metabolism and plays a crucial role in N assimilation.^7^ Photosynthesis is an important metabolic process involved in material cycling and energy transfer in plants.^8^ Both excess and inadequate N supplies influence plant N content and the photosynthetic rate^9^ and have a significant impact on leaf color and plant growth and development.^10^ In sugar beet, N management affects productivity, and reasonable N applications are beneficial to improve the quality of sugar beet.^11^ Moreover, the atmospheric N level is projected to be 150% higher in 2050 than in 2010, with the agricultural sector accounting for 60% of this increase.^12^ Enhanced-efficiency fertilizers aim to reduce nutrient losses and improve environmental and economic productivity by using the exact amount of fertilizer needed for crops and avoiding the overuse of N fertilizers. Therefore, understanding the response to N deficiency and the mechanisms of low-N (LN) tolerance is highly important for the sustainability of modern crop production.

Root structure determines the ability of plants to acquire N from the soil.^13^ The study of root system architecture can be carried out by evaluating a combination of polygenic traits involving subroot system parameterization.^14^ The nutrient efficiency of plants increases when the number of root hairs, root density, root longevity, and maximum root penetration per unit root length increase.^15^ It was found that LN effectiveness induced an increase in lateral root length, total number of roots, total root surface area and root volume for different rice genotypes.^16^ The root system topology of N-deficient seedlings has been shown to transform into a dichotomous system^17^ with short, thin, highly productive lateral roots.^18^ Understanding the response of root architecture to LN use is essential to determine the potential for sugar beet to tolerate N deficiency.

Plants can rapidly sense and respond to LN stress, which leads to oxidative stress and induces lipid peroxidation by producing reactive oxygen species (ROS) through a series of physiological and biochemical reactions, such as a decrease in chlorophyll content, an increase in related enzyme activities, and, especially, an increase in lipid peroxidation (via increased malondialdehyde (MDA) and proline (Pro) contents).^19^ Reactive oxygen includes several highly aggressive and cytotoxic chemicals, including singlet oxygen, superoxide, peroxide and hydroxyl radicals, produced by the action of light.^20^ The accumulation of ROS is tightly controlled by the antioxidant system and has the potential to alter essential biomolecules such as proteins, lipids, DNA and monosaccharides.^21^ Nadeem et al. found that under LN, toxicity caused by an overaccumulation of NH_4_^+^ in grains was neutralized by the induction of catalase (CAT), superoxide dismutase (SOD) and peroxidase (POD) activities.^22^ Zhu et al^23^ found that N deficiency led to increased lipid peroxidation and SOD activity in cannabis cell membranes. These studies suggest that various physiological regulatory mechanisms present in sugar beet may constitute an effective strategy to improve its tolerance to LN stress.

The global environmental impact can be minimized by developing crop plant varieties that are amenable to sustainable management practices and present improved N use efficiency (NUE), thus reducing the use of N fertilizer. The regulatory mechanism underlying the response to N stress in sugar beet, whose growth and sugar development and accumulation are highly restricted by N, needs to be analyzed in depth. Therefore, in this study, we mainly aimed (1) to evaluate the response of nine excellent genotypes of sugar beet to LN, as measured by their morphological and physiological changes; (2) to evaluate the NUE and N tolerance of sugar beets; and (3) to explore the significant N-tolerant genotypes and the mechanism underlying NUE for future studies on the pathways involved in NUE in sugar beets.

## 2 Materials and methods

### 2.1 Plant material, growth conditions and treatments

Nine sugar beet genotypes (94004–2 (A), 92008 feng (B), 92008–1 (C), 92017/1-4 (D), 92021-1-1 (E), 780016B/12 superior (F), 92011 (G), 92017/1-8 (H) and 92015-2-3 (I)) that were preserved in the National Beet Medium-Term Gene Bank (Harbin, China) were obtained from Heilongjiang Province. All of these genotypes presented excellent taproot yields, sucrose contents, sucrose yields and N contents. The seeds of these plants were soaked overnight in 2‰ thiram and rinsed with distilled water. After sterilization, the seeds were sown in treated vermiculite and then allowed to germinate for 8 d. When the cotyledons unfolded, seedlings of uniform size were transplanted into plastic containers (L × W × H: 37.5 × 25.5 × 13.5 cm) containing 8 L of Hoagland’s solution with a normal N supply (CK, 10 mmol·L^−1^). During the experiment, oxygen was introduced into the Hoagland solution. All seedlings were grown in an artificial climate chamber with a 14/10 h photoperiod, a 27/18°C temperature cycle, 45–55% relative humidity, and a 200 µmol·m^−2^·s^−1^ light intensity.

LN treatment (0.5 mmol·L^−1^) was adjusted via the dose of KNO_3_ and Ca(NO_3_)_2_ in the Hoagland nutrient solution. Samples were taken after one week of normal incubation and after 7 d of LN stress treatment. Afterward, whole seedlings were harvested for plant phenotypic observations and for biomass and N content determination. The leaves and roots were flash-frozen in liquid N and stored at −80°C for the determination of physiological indexes.

### 2.2 Morphological index and biomass measurements

The phenotypic characteristics of sugar beet seedlings were imaged and recorded after one week of N stress. The hypocotyl diameter, plant height (PH) and root length were measured accurately. An Epson Expression 1680 Scanner (Seiko Epson Corp., Tokyo, Japan) was used to scan the roots at 800 dpi resolution, and the total root surface and leaf area (LA) were analyzed using analysis software (WinRhizo Regent Instrument Canada INC., Quebec, Canada). The seedlings were divided into two parts, namely, aboveground and belowground parts, and rinsed with distilled water, after which the surface water was removed by blotting for fresh weight determination. The dry weight was obtained after the materials were heated at 105°C for 30 min and then dried at 80°C to a constant weight. The root-to-shoot ratio (RSR) was calculated according to the dry weight.

### 2.3 Determination of chlorophyll and soluble protein contents

Samples (0.2 g) were ground in 10 ml of 95% ethanol until the tissue was completely bleached and then centrifuged at 3000 rpm for 15 min at 4°C. A spectrophotometer (UV-1800 240 V, Shimadzu Corporation, Kyoto, Japan) at 470 nm, 665 nm and 649 nm was used for determination. The chlorophyll content (chlorophyll a+ chlorophyll b) was calculated according to Wang and Huang’s protocol.^24^ The protein content was determined by a Biosharp BCA protein concentration kit (product number BL521A), and the absorbance was measured at 562 nm by an enzyme-labeled instrument.

### 2.4 Determination of antioxidant enzyme activities, lipid peroxidation and Pro content

SOD and POD activities were determined by the nitroblue tetrazolium (NBT) method and guaiacol method, respectively.^25^ Samples were ground with phosphate buffer (pH = 7.8), and the supernatant was aspirated by centrifugation. SOD activity was measured on the basis of its absorbance at 560 nm, with 50% inhibition of the photochemical reduction of NBT representing one enzymatic unit of activity (U). POD activity was measured for 3 min, and one U was taken as the amount of enzyme that changed the absorbance at 470 nm by 0.01 in 1 min.

CAT and ascorbate peroxidase (APX) activities were determined by polyvinylpyrrolidone and ethylenediaminetetraacetic acid methods.^26^ The CAT activity assay lasted 3 min; one U was taken as the amount of enzyme that reduced the absorbance at 240 nm by 0.1 in 1 min. For APX, the total assay lasted 90s; one U was the amount of enzyme that reduced the absorbance at 290 nm by 0.01 in 1 min.

The thiobarbituric acid method was used to determine the malondialdehyde (MDA) content.^27^ A total of 0.5 g of sample tissue was ground in a prechilled mortar; then, 5% trichloroacetic acid (TCA) was added, and the homogenate was centrifuged at 3000 r/min for 10 min. The absorbance values were determined at 450 nm, 532 nm and 600 nm.

The Pro content was determined by the acidic ninhydrin method. Fresh samples (0.5 g) were ground in 3% sulfosalicylic acid solution. After centrifugation, glacial acetic acid and ninhydrin were added to the supernatant. The mixture was subsequently incubated at 100°C for 30 min for the coloration reaction; afterward, it was allowed to cool, and then toluene was added. The absorbance was recorded at 520 nm.

### 2.5 Statistical analysis

Five replications were included for determining the phenotypic indexes, and the physiological and biochemical analyses were repeated three times. The data were analyzed using SPSS statistical software (version 26.0, SPSS, Chicago, USA). To verify the significance, we performed independent sample t tests and one-way analysis of variance (ANOVA). All significance analyses were performed at the P < .05 or 0.01 level. Pearson correlation analysis (PCA) was used to assess the relationships between the different traits. To accurately assess the response of sugar beet seedlings to N deficiency, PCA was performed in conjunction with the LN/CK ratio in nine sugar beet genotypes. Finally, based on the F value of each genotype, Origin 2021 was used for systematic clustering and comprehensive evaluation of the genotypes. All the data were plotted by Origin 2021.

An affiliation function was used to generate an integrated evaluation value D to assess the LN tolerance of the different sugar beet genotypes.^28^ The relevant indicators were calculated as follows:

$X_{i}$=$\frac{Measured\;values\;of\;low\;nitrogen\;stress}{The\;measured\;value\;of\;normal\;nitrogen\;supply}$ (1)

Affiliation function value: $\mu \left(X_{i}\right)=\frac{\left(X_{i}-X_{imin}\right)}{\left(X_{imax}-X_{imin}\right)}$, (i=1, 2, …, n) (2)

where $X_{i}$ is the i-th composite index, $X_{min}$ is the minimum value of the i-th composite index, and $X_{max}$ is the maximum value of the i-th composite index.

Composite index value: $F\left(Xj\right)=a_{1j}X_{1j}+a_{2j}X_{2j}+\cdots +a_{ij}X_{ij}$ (i=1, 2, …, n;j = 1, 2, …,n) (3)

where $F\left(Xj\right)$ is the j-th composite index value, $a_{ij}$ denotes the eigenvector corresponding to the eigenvalue of each single index, and $X_{ij}$ is the standardized value of each single index.

Combined indicator weight: $w_{j}=\frac{P_{j}}{\sum_{j=1}^{n}P_{j}}$ (j = 1, 2, …,n) (4)

where $w_{j}$ denotes the weight of the j-th composite index among all composite indicators and $P_{j}$ represents the variance contribution rate of the j-th composite index for each resource.

Comprehensive index evaluation: $D=\overset{n}{\underset{j=1}{\sum }}\left[F\left(X_{j}\right)\times W_{j}\right]$ (j = 1, 2, …,n) (5)

where the D value is the comprehensive evaluation value.

## 3 Results

### 3.1 Low N stress influenced the morphology of sugar beet seedlings

**Figure 1.**
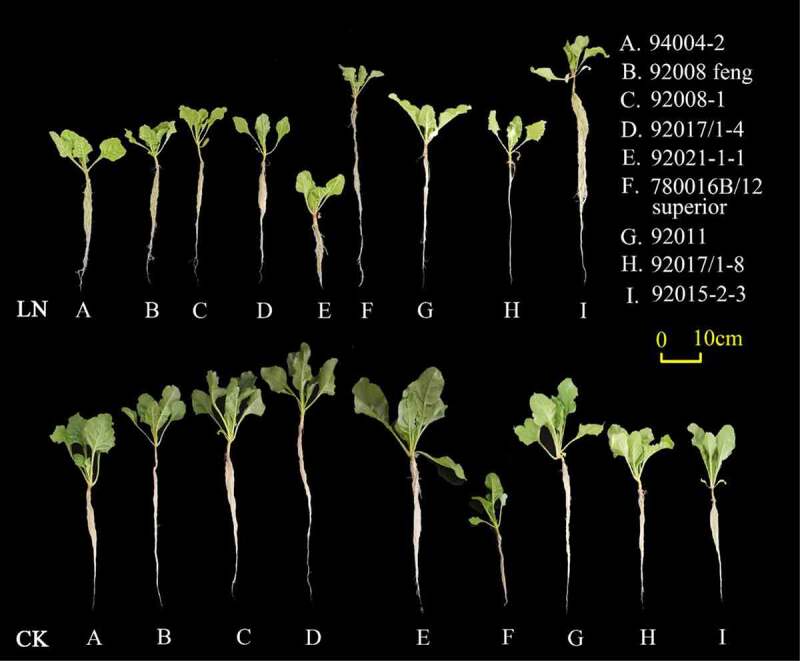
Growth and phenotype of nine sugar beet genotypes under LN stress. Low nitrogen (LN): 0.5 mmol·L^−1^ N; CK: 10 mmol·L^−1^, the same key applies to other figures and tables.

**Figure 2.**
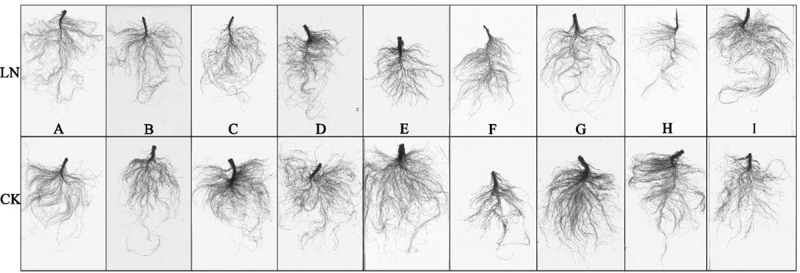
Effect of LN on the root morphology of the nine sugar beet genotypes.

**Figure 3.**
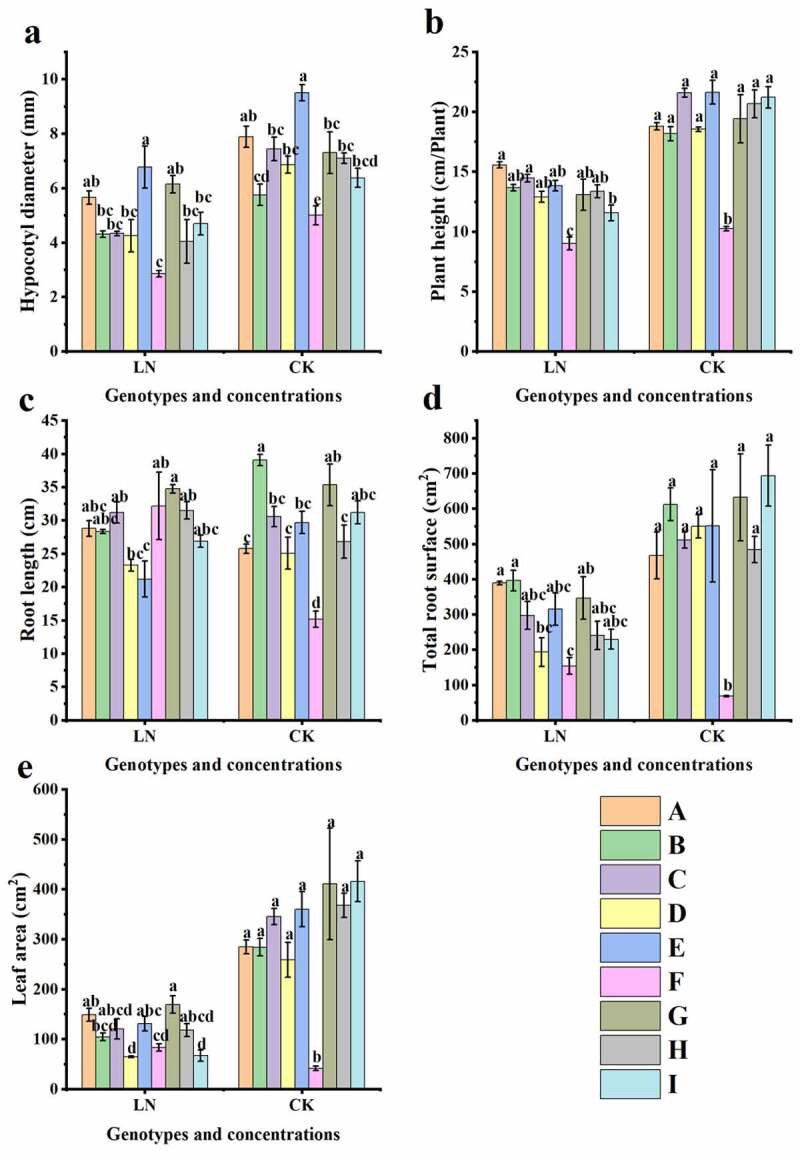
Morphological changes in the nine sugar beet genotypes under low nitrogen treatment. (a) Hypocotyl diameter, (b) plant height, (c) root length, (d) total root surface, and (e) leaf area. Lowercase letters indicate a significant difference between each genotype at the same nitrogen supply level (p < .01), as analyzed by Duncan’s multiple tests. The same key applies to other figures.

As shown in Figures 1 and 2, all nine sugar beet genotypes (A-I) exhibited different N deficiency (N0.5) symptoms at the seedling stage, such as chlorotic and thin leaves, especially in the young leaves. The adaptation of root systems to LN was similar across genotypes, presenting smaller root surface areas, fewer lateral roots, and a lower root density compared with those under normal N supply, except for 780016B/12 superior (F), which showed increased root and leaf growth under LN conditions. Further data analysis showed that LN limited the growth of most genotypes, and the morphological changes in hypocotyl diameter, plant height, root length, total root surface, and leaf area differed significantly among the nine genotypes (Figure 3 and Table S1). Among them, all tested phenotypic indexes under LN declined significantly in 92008 feng (B) (p < .05 or p < .01), but there was no significant difference in all examined indexes for 92011 (G), except for hypocotyl diameter. All the values of 780016B/12 superior (F) were lower than those of the other genotypes under LN conditions, except for root length.

### 3.2 Chlorophyll and protein content changes under LN treatment

**Figure 4.**
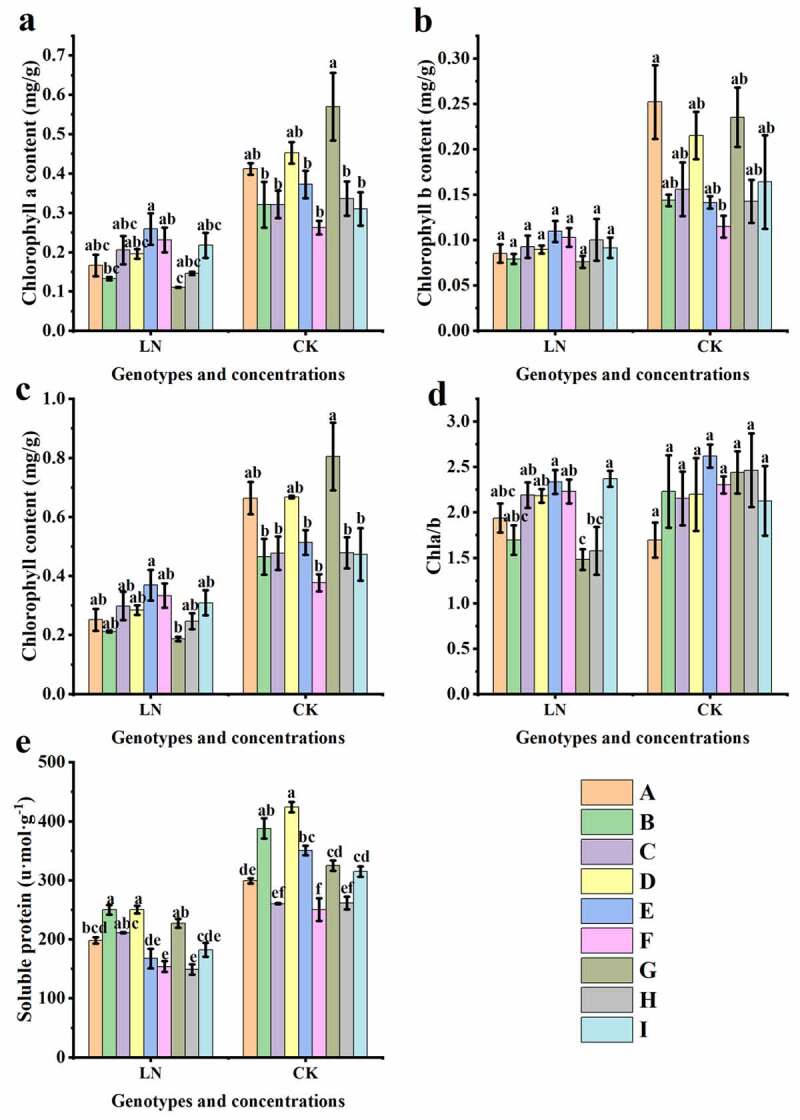
Changes in chlorophyll a content (a), chlorophyll b content (b), chlorophyll content (c), Chl a/b (d) and soluble protein (e) in different sugar beet genotypes under low nitrogen treatment.

As shown in Figure 4 and Table S2, both chlorophyll and soluble protein synthesis in sugar beets were inhibited by N deficiency, especially in 92011 (G), whose chlorophyll a, chlorophyll b, total chlorophyll content and Chl a/b were significantly reduced by 80.53%, 67.66%, 76.78% and 39.30%, respectively, compared with CK (p < .05 or p < .01). In contrast, there was no significant chlorophyll decline in 92008–1 I, 780016B/12 superior (F) and 92015-2-3 (I) under LN conditions (Table S2). Under LN conditions (Figure 4, p<.01), there were significant differences in chlorophyll and soluble protein contents between different germplasms, except for chlorophyll b; among them, 92021-1-1 (E) and 780016B/12 superior (F) had a higher chlorophyll content, and 92008 feng (B) and 92017/1-4 (D) exhibited higher soluble protein contents in general.

### 3.3 Effect of low N stress on the biomass accumulation of sugar beet seedlings

**Figure 5.**
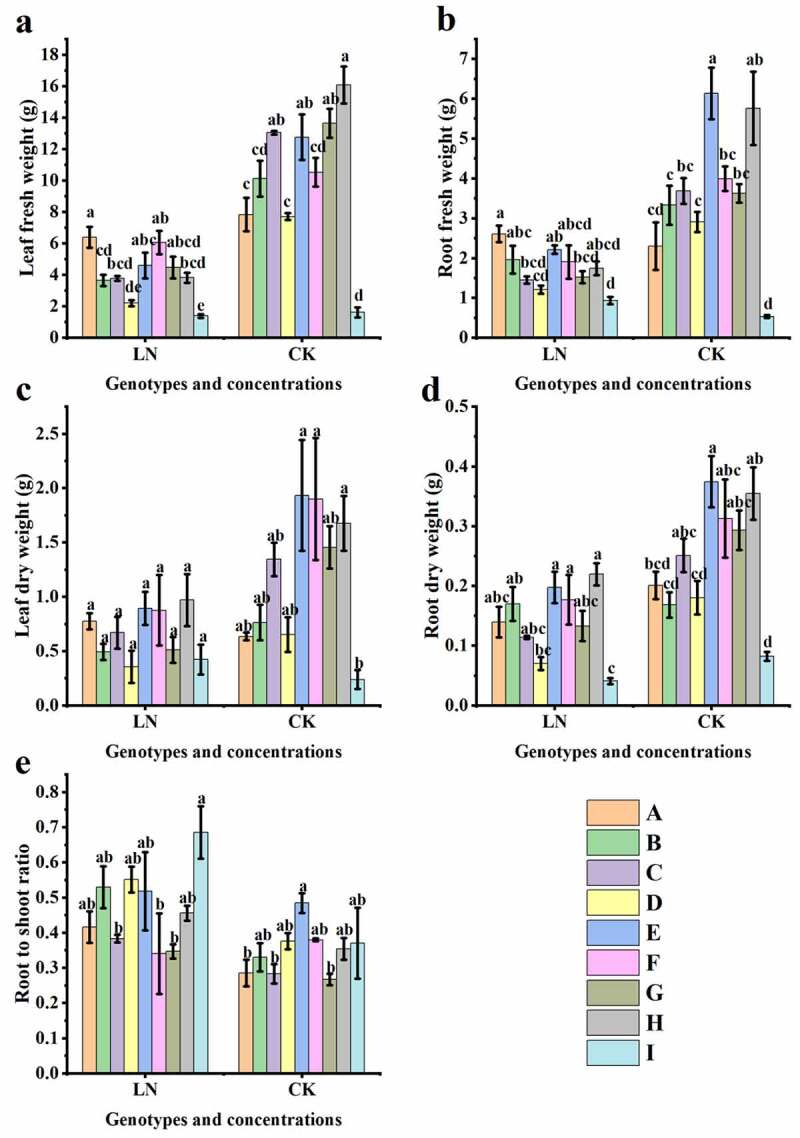
Biomass of different genotypes under low nitrogen treatment. (a) Leaf fresh weight, (b) root fresh weight, (c) leaf dry weight, (d) root dry weight, and (e) root to shoot ratio.

Under LN conditions, the biomass of each sugar beet genotype differed significantly, except for leaf dry weight (Figure 5, p<.01); 94004–2 (A) showed higher leaf and root fresh weight values, while 92015-2-3 (I) had a greater root to shoot ratio compared with the other genotypes. Although low N restricted the biomass accumulation of sugar beets to varying degrees, compared with CK, it can be seen from Table S3 (p < .05 or p < .01) that LN did not significantly influence any of the biomass indexes in 94004–2 (A). However, these indexes were affected in 92011 (G), but 780016B/12 superior (F) showed no significant differences in leaf dry weight, root dry weight and root-to-shoot ratio between LN and CK.

### 3.4 N accumulation in different sugar beet genotypes under LN conditions

**Figure 6.**
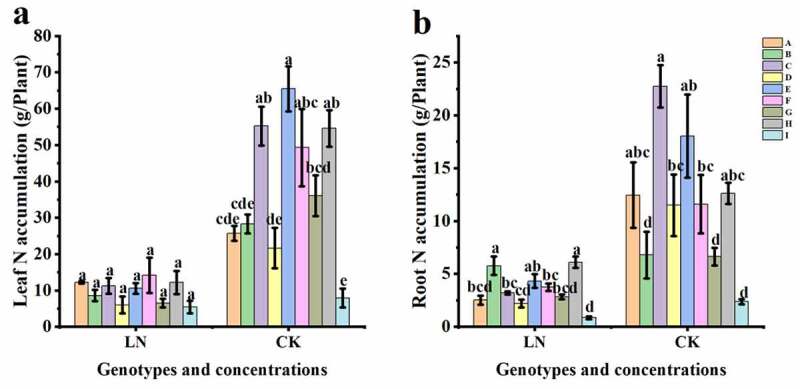
Distribution characteristics of N in nine genotypes under different nitrogen supply levels. (a) N content in leaves; (b) N content in roots.

Leaves contributed more N accumulation to sugar beet seedlings than roots (Figure 6). Under LN treatment, there was no significant difference in N accumulation in the leaves between the tested genotypes (Figure 6a, p<.01), but in the roots, the N content was significantly different (Figure 6b, p<.01). LN significantly reduced the N accumulation in both leaves and roots of 92008–1 (C), 92021-1-1 (E), 780016B/12 superior (F), 92011 (G), and 92017/1-8 (H) (Table S4, p < .05 or p < .01), but 780016B/12 superior (F) contained the highest N in leaves among the nine genotypes, indicating that this line had a higher N tolerance than the others (Figure 6a).

### 3.5 LN tolerance coefficients of sugar beets

**Figure 7.**
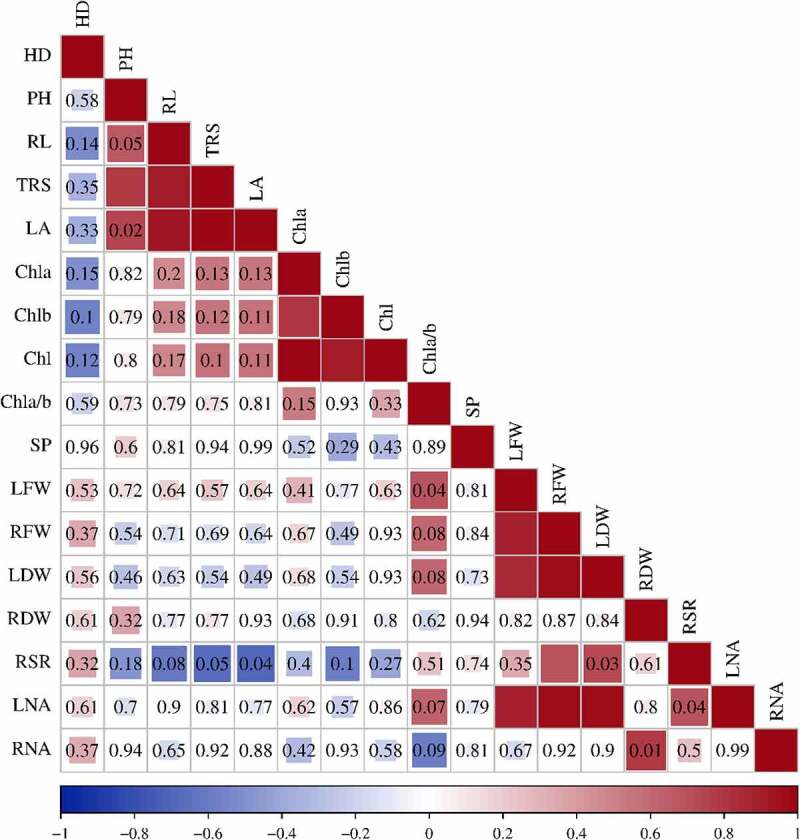
Low nitrogen tolerance of nine sugar beet genotypes by Pearson correlation coefficient (r) analysis. Hypocotyl diameter: HD; Plant height: PH; Root length: RL; Total root surface: TRS; Leaf area: LA; Chlorophyll a: Chl a; Chlorophyll b: Chl b; Chlorophyll content: Chl; Chl a and b ratio: Chl a/b; Soluble protein: SP; Leaf fresh weight: LFW; Root fresh weight: RFW; Leaf dry weight: LDW; Root dry weight: RDW; Root-to-shoot ratio: RSR; Leaf N accumulation: LNA; and Root N accumulation: RNA. The same abbreviations are used below. All the data were based on the ratio of N deficiency to N sufficiency. Scale: the bright blue to bright red colors represent negative and positive correlations, respectively. *: P < .05, **: P < .01.

To screen suitable measurements for evaluating low nitrogen stress tolerance and nitrogen accumulation capacity in different sugar beet germplasms, correlations and variation coefficients were analyzed for 17 growth-related indexes of the nine sugar beet genotypes under both LN and CK (Figure 7, Table 1). Leaf fresh and dry weight (LFW and LDW) and root dry weight (RDW) showed significant positive correlations with root N accumulation (LNA) (P < .01). Moreover, hypocotyl diameter (HD), root length (RL), chlorophyll a (Chl a), soluble protein (SP), leaf fresh weight (LFW), root fresh weight (RFW), leaf dry weight (LDW) and leaf N accumulation (LNA) showed greater variation under LN than under CK and were considered to better reflect the difference in LN tolerance between sugar beet genotypes.

**Table 1.** Variation coefficient of LN tolerance-related growth characteristics in nine sugar beet genotypes.

	LN				CK				CV(LN)-CV(CK)
	Average	Variation range	SD	CV(LN)	Average	Variation range	SD	CV(CK)	
HD	4.780	0.019–0.199	0.385	8.046	7.021	0.028–0.105	0.388	5.521	2.525
PH	13.048	0.017–0.099	0.531	4.068	18.917	0.010–0.104	0.745	3.940	0.127
RL	28.672	0.011–0.157	1.609	5.610	28.736	0.022–0.096	1.743	6.067	−0.457
TRS	284.391	0.013–0.211	34.710	12.205	507.747	0.031–0.290	64.216	12.647	−0.442
LA	111.763	0.024–0.170	11.838	10.592	307.690	0.047–0.272	33.130	10.767	−0.176
Chl a	0.185	0.009–0.174	0.021	11.287	0.373	0.036–0.181	0.040	10.701	0.586
Chl b	0.092	0.049–0.231	0.011	11.544	0.174	0.044–0.315	0.026	14.670	−3.126
Chl	0.277	0.003–0.052	0.031	11.031	0.547	0.006–0.188	0.056	10.191	0.841
Chl a/b	1.999	0.037–0.167	0.140	7.022	2.248	0.041–0.182	0.281	12.491	−5.469
SP	198.671	0.005–0.099	8.323	4.189	319.209	0.004–0.076	9.591	3.005	1.184
LFW	4.035	0.042–0.153	0.447	11.090	10.365	0.030–0.202	0.813	7.846	3.244
RFW	1.725	0.049–0.220	0.187	10.836	3.583	0.063–0.259	0.422	11.775	−0.939
LDW	0.662	0.097–0.414	0.157	23.713	1.177	0.055–0.367	0.235	20.005	3.708
RDW	0.140	0.025–0.234	0.020	14.619	0.246	0.389–1.008	0.032	13.195	1.424
RSR	0.469	0.029–0.337	0.055	11.693	0.348	0.897–1.851	0.034	9.862	1.831
LNA	9.656	0.028–0.391	2.097	21.717	38.252	0.079–0.330	5.070	13.254	8.464
RNA	3.502	0.045–0.173	0.411	11.743	11.636	0.080–0.324	2.111	18.145	−6.402

SD, standard deviation; CV, coefficient of variance.

### 3.6 Comprehensive evaluation and cluster analysis of the LN tolerance of sugar beet seedlings

To further analyze low nitrogen tolerance coefficients in nine sugar beet genotypes, four principal components with eigenvalues ≥1 were extracted because they explained more than 87.5238% of the total variance in the dataset (Table S5). Based on the PCA results, the scores of each comprehensive index were obtained, and affiliation function analysis was performed. The integrated evaluation value (D) was used as the judgment criterion to determine the low nitrogen tolerance ability of each sugar beet genotype and its corresponding rank (Table 2).^29^ Among the nine genotypes, 780016B/12 superior (F) had the highest D value, indicating its excellent LN tolerance and high genetic diversity. In contrast, the D value of 92011 (G) was the lowest, reflecting its relatively weak LN tolerance. Thus, these two germplasms were selected for further LN stress-related physiological analysis. The accuracy of the germplasm selection was also supported by a cluster analysis with a Euclidean distance of 0.4 (Fig. S1).

**Table 2.** PCA and comprehensive evaluation of the nine sugar beet genotypes.

Variety	Sugar beet genotypes	F1	F2	F3	F4	D value	Sequence
A	94004–2	−0.102	0.536	0.165	−0.053	0.547	4
B	92008 feng	−0.072	0.184	0.219	0.083	0.414	7
C	92008–1	0.175	0.266	0.013	−0.030	0.424	6
D	92017/1-4	0.007	0.231	0.027	−0.023	0.242	8
E	92021-1-1	0.208	0.249	0.013	0.080	0.549	3
F	780016B/12 superior	0.774	0.504	0.144	0.032	1.454	1
G	92011	−0.105	0.024	0.132	−0.029	0.023	9
H	92017/1-8	0.158	0.133	0.072	0.068	0.431	5
I	92015-2-3	−0.303	0.749	0.063	0.051	0.559	2

### 3.7 Physiological responses of 780016B/12 superior and 92011 to LN stress

**Figure 8.**
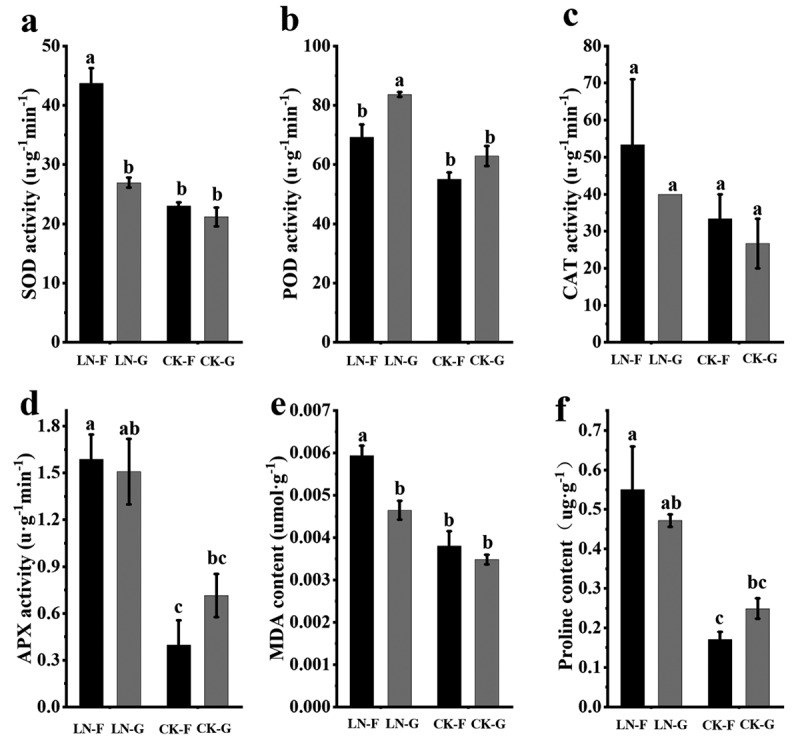
Changes in the physiological activity of LN-tolerant and LN-sensitive sugar beets under different nitrogen supply levels. (a) SOD activity, (b) POD activity, (c) CAT activity, (d) APX activity, (e) MDA content, and (f) proline content. Lowercase letters indicate a significant difference between each germplasm and nitrogen supply level (p < .01).

Low nitrogen stress differentially affected the physiological response of the low N-tolerant germplasm 780016B/12 superior (F) and the sensitive germplasm 92011 (G) by promoting antioxidant enzyme activities to varying degrees (Figure 8 a-d). There was almost no significant difference between the two genotypes under normal N supply (CK). In contrast, when suffering from low N stress, the SOD, CAT, and APX activities and proline contents of 780016B/12 superior (F) were 62.70%, 10.00%, 188.92% and 131.82% higher than those of 92011 (G), respectively. Compared with the CK condition, LN significantly triggered the activities of these enzymes and proline production in 780016B/12 superior (F); however, 92011 (G) showed no significant differences (Figure 8). In conclusion, different sugar beet genotypes showed different responses to LN stress, which once again verified the accuracy of the above results.

## 4 Discussion

Nitrogen is the most important nutrient limiting factor in sugar beet, and the growth and primary root development of sugar beet are directly related to nitrogen levels in the surrounding environment. Excellent root morphology and physiological activity under low N conditions are important features for efficient N utilization. In the present study, low N resulted in a herringbone root topology of sugar beets (Figure 2), which would be more effective in acquiring mobile nutrients with more internal chains and longer links in low nutrient zones.^30^ The LN-tolerant germplasm 780016B/12 superior (F) showed obvious increases in root length, root surface area, and leaf area and the highest N accumulation in leaves under low N stress (Figure. 1–3 and Figure 6a). It was further confirmed that N accumulation was not only dependent on root morphology but also related to the nitrogen uptake capacity of the germplasm itself.^31,32^ The adaptive changes in root morphology and physiological response may facilitate different physiological activities, thus promoting the growth of sugar beets with different levels of LN tolerance.

The selection of low N tolerance indicators varies among crops, but the response to stress depends largely on the combined effects of morphology and physiology. By comparing 19 barley germplasms, it was possible to effectively distinguish between low-nitrogen tolerant and low-nitrogen sensitive genotypes by considering relative single plant nitrogen uptake and relative nitrogen physiological utilization efficiency.^33^ In our study, through correlation and coefficient of variation analyses (Figure 7 and Table 1), it can be speculated that HD, TR, Chl a, SP, LFW, RFW, LDW and LNA are feasible indicators to evaluate the low nitrogen tolerance of sugar beets. It seems that low-nitrogen tolerance in sugar beet is a complex trait that is determined by both genetic and environmental factors. Therefore, based on the combination of low-nitrogen tolerance (D) and cluster analysis (Table 3 and Figure 8), we comprehensively evaluated nine sugar beet genotypes and identified a low nitrogen-tolerant genotype 780016B/12 superior (F) and a sensitive genotype 92011 (G) for further exploration of the LN response mechanism. The former, which was less affected by nitrogen deficiency, could ultimately be used to reduce production costs and environmental stress.

Chlorophyll is synthesized from nitrogen-containing compounds and is the most important component of chloroplasts, which are involved in photosynthesis. It has been widely reported that low nitrogen stress suppresses leaf chlorophyll content and thus the photosynthetic capacity of plants.^34^ Chl a is abundant in PSII, while Chl b is concentrated around the tentacles of the light-trapping complex.^35^ In this study, different from the LN-tolerant germplasm 780016B/12 superior (F), there was a significant difference in the Chl a content (P < .05) or Chl b content (P < .01) in the LN sensitive germplasm 92011 (G) under low N stress, compared with the control condition (Table S2). This result indicated that Chl b was more sensitive to low nitrogen than Chl a in LN-sensitive genotypes. Cystoid protein (chlorophyll) and soluble protein (rubisco) are two main forms of N storage in leaf chloroplasts, and they functionally represent the light and dark responses of photosynthesis, respectively.^23,36,37^ In this study, the reduced N accumulation in leaves (Figure 6a) under LN made the leaves yellowish or lighter green (Figure 1), and the chlorophyll content decreased, indicating that the sugar beets were suffering from stress due to inhibited nitrogen assimilation.^22,31^ Different genotypes showed various chlorophyll and soluble protein contents under low N (Table S2), which may be related to their own LN tolerance capacity and genetic characteristics. Compared with sensitive genotype 92011 (G), nitrogen deficiency did not significantly restrict chlorophyll production in the LN-tolerant genotype 780016B/12 superior (F), and an increase in the dark response of photosynthesis also increased its soluble protein content (Table S2, p < .05 or p < .01).

Photosynthesis is an important biological process that affects the accumulation of energy and dry matter in plants.^35^ Similar to what has been found for other plants,^38^ our study showed that N deficiency led to a reduction in biomass (Figure 5a-d) and an increase in the root-to-shoot ratio (Figure 5e) in sugar beet seedlings. Sugar beet seedlings transfer nutrients to the root system for growth in order to counteract nitrogen shortage. The biomass of each sugar beet genotype was differentially affected by N levels. As shown in Table S3, N deficiency significantly reduced leaf and root dry weight and the root crown ratio (P < .05), as well as leaf and root fresh weight (P < .01), in LN-sensitive 92011 (G); however, there was no significant influence on leaf and root dry weight and the root-to-shoot ratio of 780016B/12 superior (F). This may be an important difference between LN-tolerant and LN-sensitive genotypes in their response to low nitrogen supply. At present, the adaptation of beet roots regarding the aboveground parts under nitrogen starvation has not yet been explored. Here, LN-sensitive genotype showed more pronounced changes in biomass accumulation under low N stress than LN-tolerant genotype. By reducing the differences in biomass, the low nitrogen-tolerant genotype increased the root length and root surface area, and thus the area in which the root contacts the soil, which facilitated nutrient uptake by the plant and allocated more assimilated products to the roots. This is a compensatory mechanism for nutrient capture by sugar beet to adapt to nitrogen scarcity.

Reactive oxygen species (ROS) act as important secondary messengers in plants in response to various stresses.^39^ Superoxide dismutase (SOD) is the first barrier against ROS, and this enzyme changes superoxide anion radicals (O^2-^) into hydrogen peroxide (H_2_O_2_), which is subsequently neutralized by conversion to H_2_O in a reaction catalyzed by CAT or APX.^23,40,41^ A lack of or excess nitrogen can promote the formation of ROS in plants, thus affecting the integrity of cell membranes as well as chloroplasts and photosynthetic machinery; some enzymes including SOD, POD, CAT, and APX and other nonenzymatic molecules are induced to counteract oxidative stress.^42,43^ In this study, it was shown that the activities of SOD and APX were significantly higher in 780016B/12 superior (F) than in 92011 (G) under low nitrogen stress (Figure 8a, d). This kind of antioxidant defense system helps to protect photosynthetic organs from oxidative stress induced by N deficiency,^44^ indicating that these two antioxidant enzymes may be closely related to the LN tolerance capacity of 780016B/12 superior (F). However, the POD activity of 92011 (G) was much higher than that of 780016B/12 superior (F). These results are similar to the findings of Mrid et al.,^44^ and the increased POD activity in 92011 may be due to the accumulation of damage caused by prolonged stress. Proline is one of the main osmotic agents that improves membrane stability, protects protein molecules, reduces ROS formation and scavenges ROS to increase tolerance to osmotic stress.^45,46^ In the present study, we found that 780016B/12 produced much more proline than 92011 to repair cell damage due to MDA accumulation under LN stress, which in turn induced SOD and APX activities. Therefore, the relationship between the genetic background and the capacity for LN tolerance needs to be verified with genetic transformation or genome editing methods.

## 5 Conclusion

In this study, the tolerance and adaptability characteristics of different sugar beet genotypes to low nitrogen supply were comprehensive evaluated through the analysis of external morphology and internal physiological response. It was found that low nitrogen-tolerant genotypes could reduce the difference in biomass, increase root length and surface area, and then expand the contact area between roots and the soil, which was conducive to the uptake of nutrients by sugar beets and the allocation of assimilation products to the roots. This is a compensation mechanism for sugar beets to capture nutrients to adapt to nitrogen deficiency. LN-tolerant genotypes can improve the nitrogen absorption efficiency and the utilization rate of nitrogen fertilizer, thus reducing the use of nitrogen fertilizer. Thus, more sugar production can be obtained on less land. This study provides a theoretical basis for high-quality cultivation and breeding by utilization of sugar beet genotypes.

## Supplementary Material

Supplemental Material
