# Supplementary material for: Tolerance and adaptation characteristics of sugar beet (Beta vulgaris L.) to low nitrogen supply
Source: Plant Signal Behav. 2022 Dec 25;18(1):2159155. doi: 10.1080/15592324.2022.2159155 (PMC9794014; doi:10.1080/15592324.2022.2159155)
Supplement: Supplemental Material [file KPSB_A_2159155_SM5339.docx]

**Supplementary information**

**Tolerance and adaption characteristics of sugar beet (*Beta vulgaris* L.) to low nitrogen supply**

Jiajia Li^1,2^ • Xinyu Liu ^1,3^ • Qi Yao ^1,3^ • Lingqing Xu ^1,2^ • Wangsheng Li ^1,2^ • Wenbo Tan^1,2^ • Qiuhong Wang^2^ •Wang Xing ^1,2^ • Dali Liu^1,2^

1 National Beet Medium-Term Gene Bank, Heilongjiang University, Harbin, 150080, P. R. China

2 Key Laboratory of Sugar Beet Genetics and Breeding, Heilongjiang Province Common College/College of Advanced Agriculture and Ecological Environment, Heilongjiang University, Harbin, 150080, P. R. China

3 Key Laboratory of Molecular Biology, School of Life Sciences, Heilongjiang University, Harbin, 150080, P. R. China

Correspondence: Wang Xing: xyjiayou_086@163.com; Dali Liu: daliliu_hlju@163.com

**Table S1** Morphological variation of nine sugar beet genotypes under low nitrogen stress versus normal nitrogen supply

| Genotypes and Traits | A | B | C | D | E | F | G | H | I |
| --- | --- | --- | --- | --- | --- | --- | --- | --- | --- |
| Hypocotyl diameter (mm) | ** | * | ** | * | * | * | * | * | * |
| Plant height (cm/plant) | ** | ** | ** | ** | ** | n.s | n.s | ** | ** |
| Root length (cm) | n.s | ** | n.s | n.s | n.s | n.s | n.s | n.s | n.s |
| Total root surface (cm^2^) | n.s | * | ** | ** | ** | * | n.s | * | * |
| Leaf area (cm^2^) | n.s | * | ** | ** | n.s | * | n.s | * | ** |

* and * * indicate significant differences at p < 0.05 and p < 0.01, respectively; n.s represents no significant difference. A. 94004-2. B. 92008 feng. C. 92008-1. D. 92017/1-4. E. 92021-1-1. F. 780016B/12 superior. G. 92011. H. 92017/1-8. I. 92015-2-3. The same asterisks and letters are used in subsequent tables.

**Table S2** Variation in chlorophyll and protein contents in nine sugar beet genotypes under low nitrogen stress versus normal nitrogen supply

| Genotypes and Traits | A | B | C | D | E | F | G | H | I |
| --- | --- | --- | --- | --- | --- | --- | --- | --- | --- |
| Chlorophyll a content (mg/g) | ** | n.s | n.s | ** | ** | n.s | * | * | n.s |
| Chlorophyll b content (mg/g) | * | ** | n.s | ** | n.s | n.s | ** | n.s | n.s |
| Chlorophyll content (mg/g) | ** | n.s | n.s | ** | n.s | n.s | * | * | n.s |
| Chl a/b | n.s | n.s | n.s | n.s | n.s | n.s | * | n.s | n.s |
| Soluble protein (µmol·g^-1^) | ** | ** | ** | ** | ** | * | ** | ** | ** |

**Table S3** Differences in the biomass of nine sugar beet genotypes under low nitrogen stress versus normal nitrogen supply

| Genotypes and Traits | A | B | C | D | E | F | G | H | I |
| --- | --- | --- | --- | --- | --- | --- | --- | --- | --- |
| Leaf fresh weight (g) | n.s | ** | ** | ** | ** | * | ** | ** | n.s |
| Root fresh weight (g) | n.s | n.s | ** | ** | ** | * | ** | * | * |
| Leaf dry weight (g) | n.s | n.s | * | n.s | n.s | n.s | * | n.s | n.s |
| Root dry weight (g) | n.s | n.s | * | * | * | n.s | * | * | * |
| Root to shoot ratio | n.s | * | n.s | * | ** | n.s | * | ** | n.s |

**Table S4** Differences in nitrogen accumulation in different sugar beet genotypes under low nitrogen stress versus normal nitrogen supply

| Genotypes and Traits | A | B | C | D | E | F | G | H | I |
| --- | --- | --- | --- | --- | --- | --- | --- | --- | --- |
| Leaf N accumulation (g/plant) | * | ** | ** | n.s | ** | * | ** | ** | n.s |
| Root N accumulation (g/plant) | n.s | n.s | ** | * | * | * | * | ** | ** |

**Table S5** The eigenvalues and contributions of each comprehensive index.

| Items | Principal component | | | |
| --- | --- | --- | --- | --- |
|  | 1 | 2 | 3 | 4 |
| Eigenvalue | 5.9686 | 4.692 | 2.3276 | 1.8909 |
| Contribution ratio (%) | 35.1091 | 27.5998 | 13.6918 | 11.1231 |
| Cumulative contribution (%) | 35.1091 | 62.7089 | 76.4007 | 87.5238 |


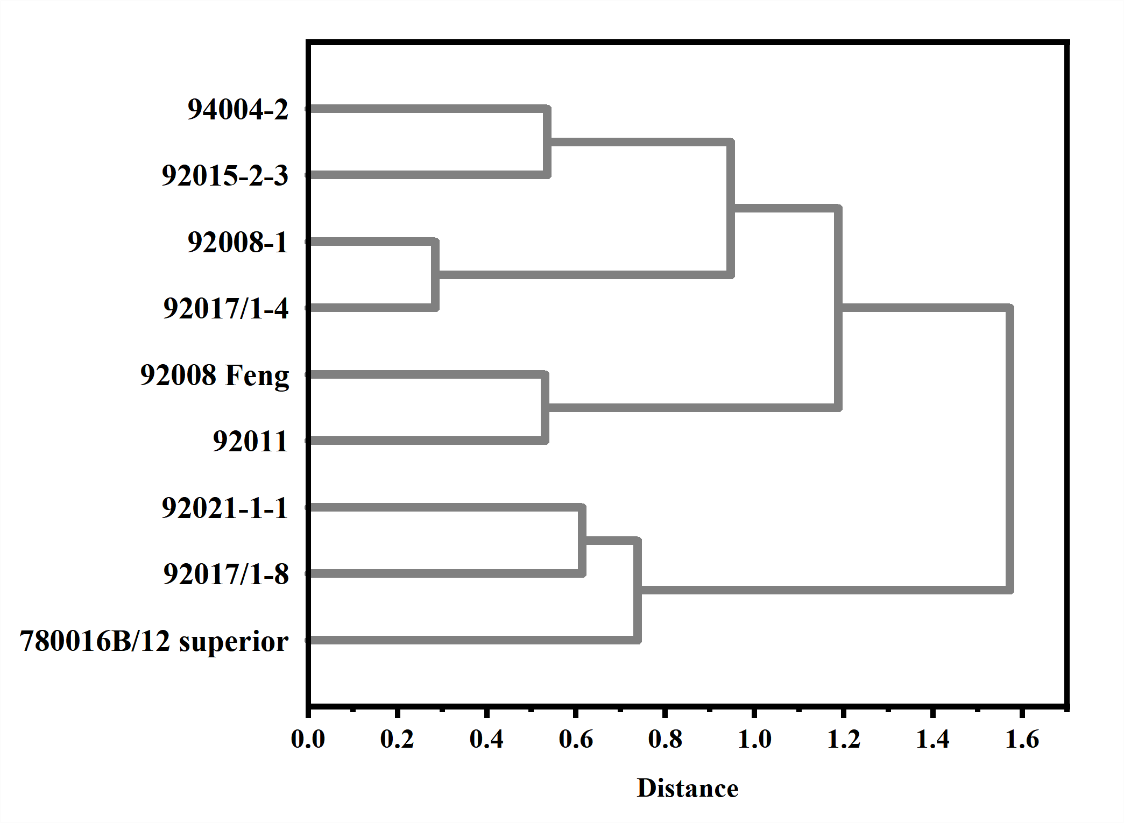


**Fig. S1** Cluster analysis of the LN tolerance of the nine sugar beet genotypes
